# Deep learning‐based image analysis reveals significant differences in the number and distribution of mucosal CD3 and γδ T cells between Crohn's disease and ulcerative colitis

**DOI:** 10.1002/cjp2.301

**Published:** 2022-11-23

**Authors:** Elin Synnøve Røyset, Henrik P Sahlin Pettersen, Weili Xu, Anis Larbi, Arne K Sandvik, Sonja E Steigen, Ignacio Catalan‐Serra, Ingunn Bakke

**Affiliations:** ^1^ Department of Clinical and Molecular Medicine (IKOM), Faculty of Medicine and Health Sciences (MH) NTNU – Norwegian University of Science and Technology Trondheim Norway; ^2^ Department of Pathology, St. Olav's Hospital Trondheim University Hospital Trondheim Norway; ^3^ Clinic of Laboratory Medicine, St. Olav's Hospital Trondheim University Hospital Trondheim Norway; ^4^ Singapore Immunology Network (SIgN) Agency for Science Technology and Research, Biopolis Singapore; ^5^ Department of Gastroenterology and Hepatology, Clinic of Medicine, St. Olav's Hospital Trondheim University Hospital Trondheim Norway; ^6^ Centre of Molecular Inflammation Research (CEMIR) NTNU Trondheim Norway; ^7^ Department of Medical Biology, Faculty of Health Sciences UiT The Arctic University of Norway Tromsø Norway; ^8^ Department of Clinical Pathology University Hospital of North Norway Tromsø Norway; ^9^ Department of Medicine, Gastroenterology Levanger Hospital, Nord‐Trøndelag Hospital Trust Levanger Norway

**Keywords:** mucosal compartments, intraepithelial lymphocytes, digital pathology

## Abstract

Colon mucosae of ulcerative colitis (UC) and Crohn's disease (CD) display differences in the number and distribution of immune cells that are difficult to assess by eye. Deep learning‐based analysis on whole slide images (WSIs) allows extraction of complex quantitative data that can be used to uncover different inflammatory patterns. We aimed to explore the distribution of CD3 and γδ T cells in colon mucosal compartments in histologically inactive and active inflammatory bowel disease. By deep learning‐based segmentation and cell detection on WSIs from a well‐defined cohort of CD (*n* = 37), UC (*n* = 58), and healthy controls (HCs, *n* = 33), we quantified CD3 and γδ T cells within and beneath the epithelium and in lamina propria in proximal and distal colon mucosa, defined by the Nancy histological index. We found that inactive CD had significantly fewer intraepithelial γδ T cells than inactive UC, but higher total number of CD3 cells in all compartments than UC and HCs. Disease activity was associated with a massive loss of intraepithelial γδ T cells in UC, but not in CD. The total intraepithelial number of CD3 cells remained constant regardless of disease activity in both CD and UC. There were more mucosal CD3 and γδ T cells in proximal versus distal colon. Oral corticosteroids had an impact on γδ T cell numbers, while age, gender, and disease duration did not. Relative abundance of γδ T cells in mucosa and blood did not correlate. This study reveals significant differences in the total number of CD3 and γδ T cells in particularly the epithelial area between CD, UC, and HCs, and demonstrates useful application of deep segmentation to quantify cells in mucosal compartments.

## Introduction

Inflammatory bowel diseases (IBDs), including ulcerative colitis (UC) and Crohn's disease (CD), are common, immune‐mediated diseases [1]. Improved knowledge in pathobiology has led to new treatments and made these diseases more manageable [2]. Histology has been increasingly important in assessing disease activity and defining remission. Scoring systems that combine histological features of activity and chronicity have been developed, mainly for clinical trials [3, 4, 5, 6]. Activity is simply defined as neutrophil infiltration, while chronic inflammation involves alterations of different cell types [7].

Difficulties in making quantitative assessments have hampered the use of histology in IBD research. Artificial intelligence (AI) applications in pathology enable the extraction of complex visual data in whole slide images (WSIs) and may reveal patterns of inflammation hidden to the human eye.

The intestinal immune defence is shaped by interactions between microorganisms, epithelial cells, and immune cells [8]. Disturbances in T lymphocyte (T cell) responses in IBD create a pro‐inflammatory microenvironment where the intraepithelial lymphocytes (IELs) are centrally located. Despite this, little is known about their role in IBD [9, 10]. The IELs are tissue‐resident T cells that broadly can be divided in a large group expressing the αβ T cell receptor, and a smaller group mainly expressing the γδ T cell receptor [8, 9, 11, 12, 13]. The γδ T cells are enriched in mucosal tissues where they patrol the epithelium, protect against pathogens, and promote epithelial repair [14, 15, 16, 17, 18, 19, 20, 21]. Their role in IBD is unclear. Human studies have shown different numbers of γδ T cells in normal and inflamed mucosa but use samples from various parts of the intestine and flow cytometric methods that do not distinguish between mucosal compartments [9, 22, 23]. Manual counting of IELs and/or γδ T cells on tissue sections has also been done [24, 25], but is subjective and prone to error.

Computational pathology uses deep learning techniques on WSIs but has not often been applied in IBD [26, 27, 28]. Our aim was to use AI‐based tools to objectively quantify and compare CD3 and γδ T cell numbers in colon mucosal compartments in IBD in a cohort of CD, UC, and healthy controls (HCs). Quantifications were made intraepithelially, subepithelially, and in lamina propria in histologically active and inactive disease, characterised by the Nancy histological index. WSIs from proximal and distal colon mucosa were included to adjust for locational variations. The impact of oral corticosteroids, age, gender, and disease duration was assessed, and correlation between γδ/CD3 cell ratios in blood and mucosa was performed. This study shows the utility of AI‐based image analysis in mapping colon mucosal immune cells, and sheds light on the IELs and γδ T cells in IBD.

## Materials and methods

### Ethics

The study was conducted in adherence to the Declaration of Helsinki and approved by Central Norway Regional Committee for Medical and Health Research Ethics (reference numbers 5.2007.910 and 2013/2013 REK Midt). Written informed consent was provided by all participants.

### Patient material

Samples were collected from an in‐house biobank of patients undergoing colonoscopy and clinical examination at St. Olav's University Hospital (Trondheim, Norway) during 2007–2018, and confirmed to have UC or CD. HCs were patients who underwent colonoscopy without significant findings. Biopsies from healthy and inflamed colon mucosa were formalin‐fixed, stored in paraffin‐embedded tissue blocks, cut into 4 μm sections, and subjected to haematoxylin and eosin (H&E) staining or immunohistochemistry (IHC). We analysed 83 biopsies from 58 UC patients, 57 biopsies from 37 CD patients, and 41 biopsies from 33 HCs. Mayo clinical and endoscopic scores were recorded for UC, and Harvey‐Bradshaw index and simple endoscopic score for CD. Patient demographics and biopsy data are summarised in Table 1.

**Table 1:** Clinical and histopathological data for all patients and samples in the cohort

Patient characteristics	UC		CD		HC
Number of patients, *n* (female/male)	58 (25/33)		37 (26/11)		33 (19/14)
Age, year, median (range)	37 (18–69)		30 (18–77)		43 (17–79)
Disease duration, month, median (range)	102 (0–480)		108 (2–564)		–
Disease extent^†^, *n*					–
UC/CD				
Non	13		3		
Proctitis/ileal	13		11		
Left‐sided (right)/colonic	13 (1)		12		
Pancolitis/ileocolonic	18		11		
Endoscopic score, *n*					–
Mayo/SES‐CD (resected)^‡^				
0/remission (0–2)	13		6 (1)		
1/mild (3–6)	21		9 (1)		
2/moderate (7–15)	17		18 (8)		
3/severe (>15)	7		4		
Clinical evaluation, *n*					–
Partial Mayo/HB index				
Remission (0–1)/(<5)	27		15^$^		
Mild (2–4)/(5–7)	16		11		
Moderate (5–6)/(8–16)	5		8		
Severe (7–9)/(>16)	10		3		
Disease specific medication, *n*					–
None	11		11		
5‐ASA/steroids [rectal]	9		0		
X‐ASA [oral]	43		8		
Steroids [oral]	14		15		
Azathioprine	6		10		
TNF inhibitors	1		4		

Biopsy characteristics	UC N0‐1	UC N2‐4	CD N0‐1	CD N2‐4	HC
Total number of biopsies, *n*	39	44	35	22	41
Biopsy localisation*, *n*					
Proximal colon	30	12	24	9	30
Distal colon	9	32	11	13	11
Nancy histological index, *n*					
0	32	–	34	–	40
1	7	–	1	–	1
2	–	12	–	6	–
3	–	19	–	7	–
4	–	13	–	9	–

CD, Crohn's disease; HB index, Harvey‐Bradshaw clinical index; HC, healthy control; N0‐1, Nancy grade 0–1; N2‐4, Nancy grade 2–4; SES‐CD, simple endoscopic score for Crohn's disease; TNF, tumour necrosis factor; UC, ulcerative colitis; X‐ASA, 5‐aminosalicylic acid (5‐ASA) and/or sulfasalazine.

* Proximal colon: coecum, ascending colon, hepatic flexure; distal colon: splenic flexure, descending colon, sigmoid colon, rectum.

^†^ At time of biopsies.

^‡^ Number of incomplete ‘at least scores’ due to bowel resection.

^$^ Incomplete value for one patient due to ostomy.

Heparinised venous blood samples were taken, and peripheral blood mononuclear cells (PBMCs) isolated by centrifugation on Lymphoprep mononuclear cell separation medium (Axis‐Shield, Oslo, Norway). The PMBCs were kept frozen on liquid nitrogen with human group AB+ serum and 10% dimethyl sulfoxide until analyses.

### Histology, IHC, and imaging

Histological scoring was performed on H&E stained WSIs by a gastrointestinal pathologist (ESR) using the Nancy histological index, a three‐descriptor system with five grades of inflammation: no or mild chronic (grade 0), moderate to severe chronic (grade 1), focally acute with few neutrophils (grade 2), moderate to severe with many neutrophils (grade 3), and ulceration with fibrinopurulent exudate and/or granulation tissue (grade 4) [29, 30]. The Nancy index was developed and validated for UC but was also used for CD here. For statistical analysis, we dichotomised the score into Nancy grade 0–1 (absence of neutrophils) and grade 2–4 (presence of neutrophils in lamina propria and/or in the epithelium). This gave five study groups: HCs with Nancy grade 0–1 (HC), inactive UC with Nancy grade 0–1 (UC N0‐1), inactive CD with Nancy grade 0–1 (CD N0‐1), active UC with Nancy grade 2–4 (UC N2‐4), and active CD with Nancy grade 2–4 (CD N2‐4). The terms active and inactive disease in this paper refer to the presence or absence of neutrophils in mucosa.

Sections underwent IHC pre‐treatment with quenching of endogenous peroxidase and antigen retrieval by 15 min boiling in Tris EDTA pH 9 buffer in a microwave oven. Primary antibodies were mouse anti‐human CD3 (M7254, clone F7.2.38, Dako Agilent, CA, USA) and mouse anti‐human TCRδ (sc‐100 289, clone H‐41, Santa Cruz, CA, USA) diluted 1:50 and 1:200 in Tris‐buffer with 0.025% Tween‐20 and 1% BSA. Incubations were performed overnight at 4 °C and immunoreactions visualised with the secondary antibody rabbit/mouse EnVision‐HRP/DAB+ kit (K5007, Dako Agilent), and counterstaining with haematoxylin. Omission of the primary antibody was negative control, and lymph node and spleen positive controls.

All sections were scanned (×40 resolution) on a NanoZoomer S360 (Hamamatsu, Hamamatsu City, Japan), and read in NDPview2 (U12388‐01, Hamamatsu). Images were captured in NDPview2 or screenshot from QuPath and further cropped in Photoshop (Adobe Photoshop CC, 20.0.6 Release). There were no systematic group differences in the orientation of biopsies.

### Flow cytometry of PBMCs

PBMCs were stained with the antibodies anti‐human CD3 (clone UCHT1, BD Biosciences, Oslo, Norway) and anti‐human TCRγ/δ (clone 11F2, BD Biosciences) for 20 min in the dark at 4 °C in PBS with 5% FBS and 2 mM EDTA (FACS buffer), washed twice with FACS buffer before re‐suspended in 100 μl FACS buffer. Samples were acquired using BD FACSymphony flow cytometer using automatic compensations.

### Deep learning‐based segmentation of the epithelium and delineation of a subepithelial zone

**Figure 1:**
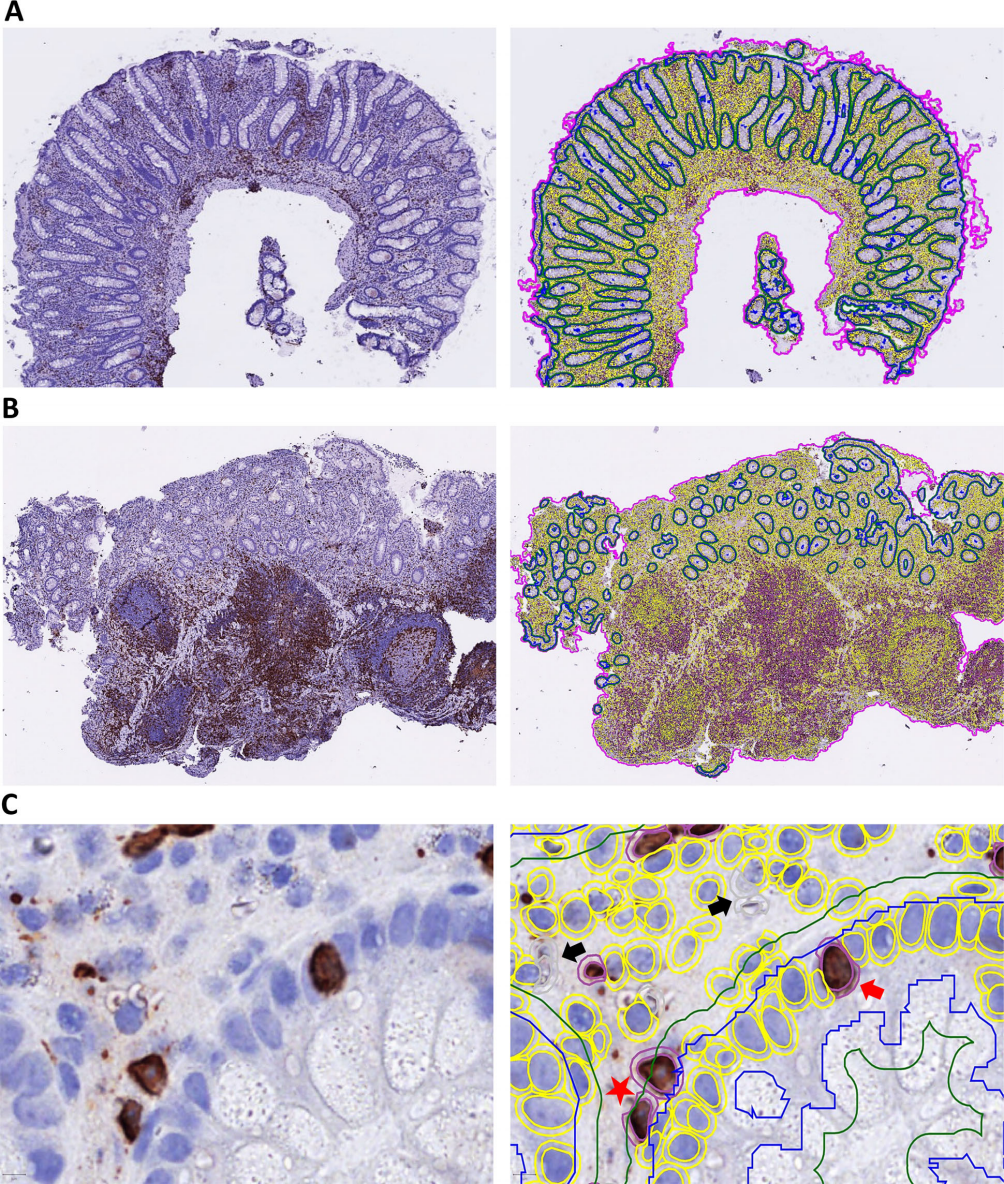
Automated deep learning‐based segmentation of colon mucosal compartments and counting of T cells. Parallel images showing CD3‐stained WSIs (brown colour) from (A) inactive normal mucosa and (B) active IBD mucosa either unannotated (left panels) or annotated in QuPath (right panels), with the epithelial and 5 μm extended subepithelial segmentation lines (blue and green) and the counted T cells (yellow circles) within the total area of the biopsies (purple line). (C) Higher magnification of parallel unannotated and annotated images showing example of segmented epithelium (blue line) and subepithelium (green line), and classification and automated counts of stained CD3 T cells (double red circles) and total cell number (double yellow circles) done in the different mucosal compartments. Examples of CD3 T cells counted in the epithelium (red arrow), in the subepithelium (red star) or artefacts that were not counted (black arrows) are indicted.

Open‐source software was used to create a pipeline for deep learning‐based epithelial segmentation [31]. In brief, the epithelium in 30 WSIs was annotated in QuPath (ESR) [32]. Labelled patches were exported to DeepMIB in Microscopy Image Browser (MIB) [33]. Two deep segmentation networks, U‐Net and SegNet, were trained based on the annotated images. Unseen WSIs were predicted in DeepMIB [34], corrected in QuPath, and the dataset gradually expanded through active learning. To delineate a subepithelial compartment with immune cells located immediately beneath the basement membrane, the QuPath functionality ‘Expand annotation 5 μm’ was used. All epithelium and subepithelium in each CD3 and γδ T cell stained WSIs were segmented (Figure 1).

### Automated quantification of CD3 and γδ T cells

To quantify CD3 and γδ T cell numbers in mucosal compartments, StarDist was called by scripting in QuPath [32, 35]. Colour deconvolution of stains was manually set for a representative image for each stain. Cell nucleus detection with StarDist was performed on a QuPath (0.2.3) Tensorflow build with the pretrained model ‘HE_heavy_augment’ using settings: requested pixel size 0.225 μm; threshold 0.01; cell expansion 1.5 μm; measure intensity. Cell nucleus detections with areas above and below 125 pixel^2^ and 3,000 pixel^2^ were excluded. A three class (IHC positive cell, IHC negative cell, and artefact) random trees object classifier (QuPath 0.2.3 tool ‘Object classification’) was trained by a pathologist (HPSP) by annotating in QuPath ~7,000 StarDist nucleus detection objects in ~30 WSIs as either IHC positive cells, IHC negative cells, or artefacts/false positive detections. Two pathologists (ESR and HPSP) agreed upon the minimum size of the cells to be counted. This was done until visually satisfactory cell classification performance was achieved on unseen slides, as assessed by the two pathologists, and the same three class random trees classifier was applied to all the CD3 and γδ T cell stained WSIs (Figure 1). Quantifications of all three cell classes from the epithelium, epithelium including the 5 μm subepithelial zone, and the whole mucosa, were made. The IHC positive and total cell numbers were exported to excel by the export measurements functionality in QuPath and imported to SPSS. Subepithelial CD3 and γδ T cell numbers were calculated by subtracting the numbers in the epithelium from the numbers in the 5 μm expanded epithelial segmentation. The numbers in lamina propria were calculated by subtracting the 5 μm expanded epithelium counts from the whole mucosa measurements. Intraepithelial numbers of CD3 and γδ T cells are presented per 100 epithelial cell which is the most common way of presenting IELs in pathology. For simplicity, the subepithelial lymphocytes are presented similarly, while CD3 and γδ T cell numbers in lamina propria are given per mm^2^ mucosa.

### Statistics

All data analysis were performed in SPSS (IBM, SPSS Statistics, v27‐28). A linear mixed model was made. We chose this method due to issues of dependencies and different sample sizes. The intraclass correlation coefficient showed moderate correlation between measurements within the same groups, justifying the choice of method. Diagnosis, Nancy grade, and colon location were fixed factors, and subjects were random factor. Numbers of CD3 and γδ T cells, and ratio of γδ/CD3 were dependent variables. Age, gender, disease duration, and oral corticosteroids were included as fixed factors in separate analyses. Normality tests with histograms showed right skewed distributions for nearly all cell measurements. Log‐transformed data displayed normal distribution and were therefore used. The residuals of the analyses displayed normal distribution. *Post hoc* tests were set to least significant difference. The significance level was set to 0.05. The tables present relative fold changes (FCs) in the number of γδ T and CD3 cells between the groups, with 95% confidence intervals (CIs). The numbers are back‐transformed, but *P* values are based on log‐transformed data. Interaction analyses between fixed factors and outcome variables were also performed. Correlations between the γδ/CD3 ratios in peripheral blood and in mucosal compartments were analysed with Spearman correlation coefficient. Comparisons between proximal and distal colon mucosal CD3 and γδ T cell numbers in inactive IBD and HC were done using the Kruskal–Wallis test. Graphs in the figures are created in GraphPad (Prism 9.1.2).

## Results

### Clinical and histopathological data

The clinicopathological data are summarised in Table 1. The CD patients were fewer, slightly younger, and more often female than the UC patients, and a higher proportion used immunosuppressant drugs (oral corticosteroids, azathioprine, and anti‐TNF). Distal colon biopsies had more active disease than proximal biopsies. Most histologically inactive biopsies had Nancy grade 0. Grades 2, 3, and 4 were equally distributed in the active groups. Ethnicity, disease extent, endoscopic, and clinical scores did not differ between groups.

### Automated deep learning‐based models give pathologist‐level segmentation, and objective cell counts in mucosal compartments

**Figure 2:**
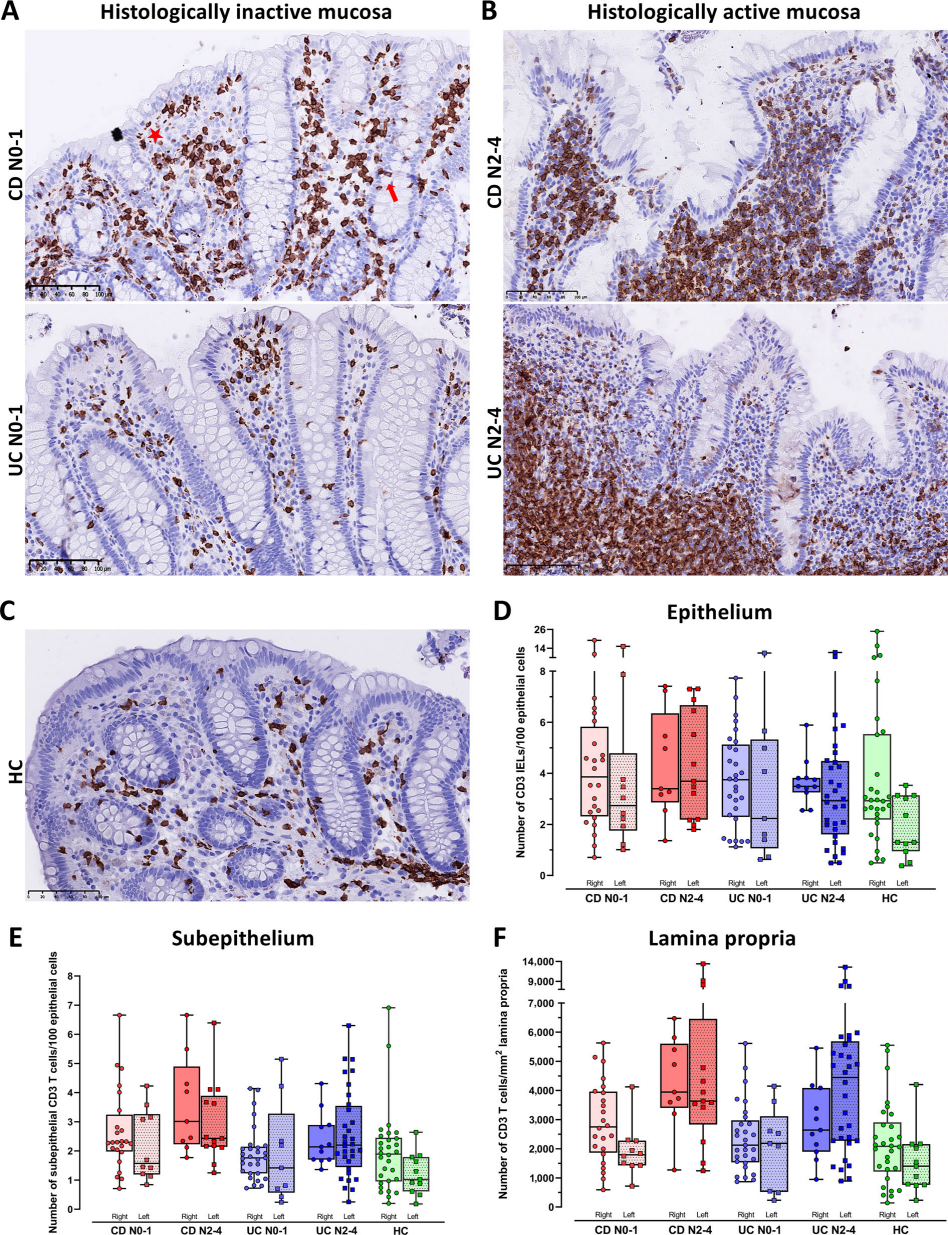
Distribution of CD3 T cells in colonic mucosal compartments of HC, CD, and UC with histologically inactive and active disease. (A–C) Representative images of colon mucosa showing the number and distribution of CD3 T cells in the epithelium, subepithelium, and lamina propria of (A) histologically inactive mucosa (Nancy score 0–1) in Crohn's disease (CD N0‐1) and in ulcerative colitis (UC N0‐1), (B) histologically active mucosa (Nancy score 2–4) in Crohn's disease (CD N2‐4) and in ulcerative colitis (UC N2‐4), and (C) in healthy controls (HC). Scale bars 100 μm. Examples of IELs and subepithelial CD3 T cells are marked with red arrow and red star, respectively, in (A). (D–F) Box and whisker plots showing median and individual numbers of CD3 T cells in biopsies from the right and left side of colon measured by computational pathology in: (D) epithelium, (E) subepithelium, and (F) lamina propria in Crohn's disease with histologically inactive (CD N0‐1) and active (CD N2‐4) mucosa, in ulcerative colitis with histologically inactive (UC N0‐1) and active (UC N2‐4) mucosa, and in HCs. Numbers for the epithelium and subepithelium are given per 100 epithelial cells and numbers for lamina propria are given per mm^2^.

**Figure 3:**
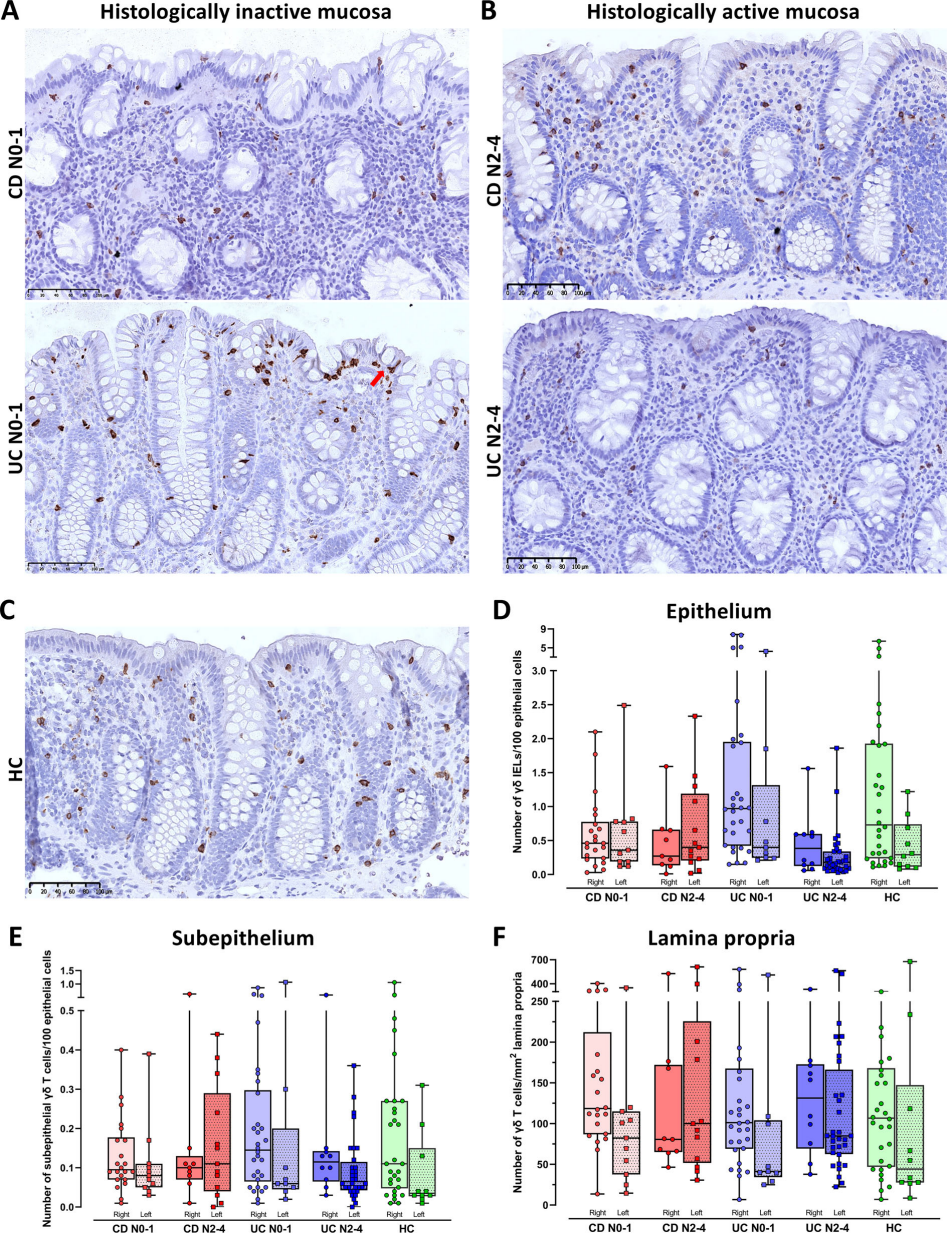
Distribution of γδ T cells in colonic mucosal compartments of HC, CD, and UC with histologically inactive and active disease. (A–C) Representative images of colon mucosa showing the number and distribution of γδ T cells in the epithelium, subepithelium, and lamina propria of (A) histologically inactive mucosa (Nancy score 0–1) in Crohn's disease (CD N0‐1) and in ulcerative colitis (UC N0‐1), (B) histologically active mucosa (Nancy score 2–4) in Crohn's disease (CD N2‐4), in ulcerative colitis (UC N2‐4), and (C) in HCs. Scale bars 100 μm. An example of a γδ IEL is marked with a red arrow in (A). (D–F) Box and whisker plots showing median and individual numbers of γδ T cells in biopsies from the right or left side of colon measured by computational pathology in: (D) epithelium, (E) subepithelium, and (F) lamina propria in Crohn's disease with histologically inactive (CD N0‐1) and active (CD N2‐4) mucosa, in ulcerative colitis with histologically inactive (UC N0‐1) and active (UC N2‐4) mucosa, and in HCs. Numbers for the epithelium and subepithelium are given per 100 epithelial cells and numbers for lamina propria are given per mm^2^.

As previously reported, our computational model displayed epithelial segmentation with pathologist‐level accuracy and delineated the epithelial and subepithelial compartments robustly, although the prediction accuracies were somewhat lower for biopsies with active disease (Figure 4 in [31]) (Figure 1A,B). We used StarDist for nucleus detection and trained a three class random trees classifier (IHC positive cell, IHC negative cell, and artefact) in QuPath for automated quantifications in the segmented compartments (Figure 1C). Variations in WSI quality created challenges that required more annotations before good models were achieved. We found inter‐individual variations in the number of CD3 (Figure 2) and γδ T cells (Figure 3), but still significant differences between subgroups, i.e. CD versus UC, active versus inactive, and proximal versus distal colon.

### CD3 and γδ T cells in mucosal compartments in inactive IBD

#### Inactive CD has fewer γδ IELs than inactive UC

We first assessed the baseline values of CD3 (Figure 2) and γδ IELs (Figure 3) in HC and inactive IBD, and the relative differences between the groups (Table 2). HC had median (95% CI) CD3 number of 2.8 (2.4–3.1) (Figure 2C,D) and γδ T number of 0.6 (0.3–1.2) (Figure 3C,D) per 100 epithelial cells. CD tended to have more CD3 and less γδ IELs than HC, while UC had similar CD3 and more γδ IELs. However, CD had significantly lower and only half the number of γδ IELs (*p* = 0.007) than UC (Table 2, Figures 2 and 3A,C,D).

**Table 2:** Relative differences in the median numbers of CD3, γδ T cells, and γδ/CD3 ratios in colonic mucosal compartments of histologically inactive mucosa. Comparisons of CD3 and γδ T cell numbers and γδ/CD3 T cell ratios in colon mucosa epithelium (IEL), subepithelium (5 μm below the basement membrane), and lamina propria, between Crohn's disease with Nancy grade 0–1 (CD N0‐1, *n* = 35), ulcerative colitis with Nancy grade 0–1 (UC N0‐1, *n* = 39), and healthy controls (HC, *n* = 41). Relative differences in median numbers are given as fold change (FC), 95% confidence interval (CI), and *P* value, where statistical significance within each row is marked with italic bold.

Histologically inactive mucosa	IEL			Subepithelium			Lamina Propria		
	FC	95% CI	*P* value	FC	95% CI	*P* value	FC	95% CI	*P* value
CD3 T cells									
CD N0‐1 versus HC	1.4	1.0–1.9	0.06	1.6	1.2–2.2	***0.001***	1.5	1.1–2.1	***0.016***
UC N0‐1 versus HC	1.2	0.9–1.7	0.29	1.2	0.9–1.5	0.30	1.2	0.9–1.6	0.25
CD N0‐1 versus UC N0‐1	1.2	0.8–1.6	0.41	1.4	1.0–1.9	***0.024***	1.2	0.9–1.7	0.19
γδ T cells									
CD N0‐1 versus HC	0.7	0.4–1.2	0.16	1.0	0.6–1.7	1.00	1.3	0.8–2.0	0.32
UC N0‐1 versus HC	1.4	0.9–2.4	0.18	1.3	0.8–2.3	0.27	1.0	0.7–1.6	0.83
CD N0‐1 versus UC N0‐1	0.5	0.3–0.8	***0.007***	0.8	0.5–1.3	0.26	1.2	0.8–1.8	0.39
γδ/CD3 T cell ratio									
CD N0‐1 versus HC	0.5	0.3–0.9	***0.017***	0.7	0.4–1.1	0.10	1.0	0.7–1.7	0.89
UC N0‐1 versus HC	1.3	0.8–2.1	0.35	1.2	0.8–2.0	0.40	1.0	0.7–1.6	0.85
CD N0‐1 versus UC N0‐1	0.4	0.3–0.7	***0.001***	0.5	0.3–0.9	***0.015***	1.0	0.6–1.5	0.97

For γδ/CD3 ratios, inactive CD had median (95% CI) ratio of only 14.4% (7.0–23.6), in contrast to 24.4% (18.5–34.8) and 30.9 (20.7–42.3) in HC (*p* = 0.017) and UC (*p* = 0.001) (Table 2). These differences became more pronounced if only patients with negative endoscopic scores were included, but the number of samples was then too small for conclusions (data not shown). Overall, inactive CD had more CD3 IELs, less γδ IELs, and significantly lower γδ/CD3 IELs ratio than HC and inactive UC.

#### Inactive CD has more CD3 cells in subepithelium and lamina propria than inactive UC and HC, but equal numbers of γδ T cells

Inactive CD had significantly more CD3 cells subepithelially and in lamina propria than HC (*p* = 0.001, *p* = 0.016), while UC and HC had equal numbers. CD also had more subepithelial CD3 cells than UC (*p* = 0.024), but equal numbers in lamina propria (Table 2, Figure 2A‐C,E,F). There were no differences in the subepithelial or lamina propria numbers of γδ T cells between CD, UC, and HC (Table 2, Figure 3A‐C, E,F). The γδ/CD3 ratios were also equal in lamina propria. Due to more subepithelial CD3 cells, inactive CD had lower γδ/CD3 ratio beneath the epithelium than UC (*p* = 0.015), with median (95% CI) 3.7% (2.8–6.1) compared to 8.8% (5.8–12.6) in UC. Overall, inactive CD had more CD3 cells subepithelially and in lamina propria than inactive UC and HC, but similar numbers of γδ T cells in these compartments.

### CD3 and γδ T cells in mucosal compartments in active IBD

#### Active CD has constant numbers while active UC has massive loss of γδ IELs

We then examined the impact of disease activity on the number of CD3 (Figure 2) and γδ T cells (Figure 3), and the relative differences between the groups (Table 3). We found no significant change in CD3 IELs in active compared to inactive disease in CD or UC. Active CD had more CD3 IELs (*p* = 0.009) than HC, while the number was the same in UC and HC (Table 3, Figure 2A–D).

**Table 3:** Relative differences in the median numbers of CD3, γδ T cells, and γδ/CD3 ratios in colonic mucosal compartments of histologically active mucosa. Comparisons of CD3 and γδ T cell numbers and γδ/CD3 T cell ratios in colon mucosa epithelium (IEL), subepithelium (5 μm below the basement membrane), and lamina propria, between Crohn's disease with Nancy grade 2–4 (CD N2‐4, *n* = 22) or Nancy grade 0–1 (CD N0‐1, *n* = 35), ulcerative colitis with Nancy grade 2–4 (UC N2‐4, *n* = 44) or Nancy grade 0–1 (UC N0‐1, *n* = 39), and healthy controls (HC, *n* = 41). Relative differences in median numbers are given as fold change (FC), 95% confidence interval (CI), and *P* value, where statistical significance within each row is marked with italic bold

Histologically active mucosa	IEL			Subepithelium			Lamina propria		
	FC	95% CI	*P* value	FC	95% CI	*P* value	FC	95% CI	*P* value
CD3 T cells									
CD N2‐4 versus HC	1.7	1.1–2.5	***0.009***	2.3	1.7–3.2	***<0.001***	2.7	1.9–3.9	***<0.001***
UC N2‐4 versus HC	1.3	0.9–1.8	0.11	1.8	1.3–2.4	***<0.001***	2.3	1.7–3.2	***<0.001***
CD N2‐4 versus CD N0‐1	1.2	0.8–1.8	0.32	1.4	1.0–2.0	***0.046***	1.8	1.3–2.6	***0.002***
UC N2‐4 versus UC N0‐1	1.1	0.8–1.5	0.59	1.5	1.1–2.1	***0.005***	1.9	1.4–2.7	***<0.001***
CD N2‐4 versus UC N2‐4	1.3	0.9–1.9	0.20	1.3	1.0–1.6	0.11	1.2	0.8–1.7	0.38
γδ T cells									
CD N2‐4 versus HC	0.6	0.3–1.0	0.06	1.2	0.7–2.2	0.55	1.4	0.9–2.4	0.15
UC N2‐4 versus HC	0.4	0.3–0.7	***0.002***	1.1	0.6–1.9	0.75	1.4	0.9–2.2	0.18
CD N2‐4 versus CD N0‐1	0.8	0.5–1.5	0.51	1.2	0.7–2.1	0.52	1.1	0.7–1.8	0.56
UC N2‐4 versus UC N0‐1	0.3	0.2–0.5	***<0.001***	0.8	0.5–1.3	0.41	1.3	0.9–1.9	0.20
CD N2‐4 versus UC N2‐4	1.3	0.7–2.3	0.36	1.1	0.6–1.9	0.75	1.1	0.7–1.7	0.83
γδ/CD3 T cell ratio									
CD N2‐4 versus HC	0.4	0.2–0.7	***0.001***	0.7	0.4–1.3	0.28	0.6	0.4–1.1	0.09
UC N2‐4 versus HC	0.3	0.2–0.5	***<0.001***	0.5	0.3–0.9	0.20	0.6	0.4–0.9	***0.020***
CD N2‐4 versus CD N0‐1	0.7	0.4–1.3	0.26	0.9	0.5–1.6	0.68	0.6	0.4–1.0	***0.037***
UC N2‐4 versus UC N0‐1	0.2	0.2–0.4	***<0.001***	0.4	0.3–0.7	***0.001***	0.5	0.4–0.8	***0.004***
CD N2‐4 versus UC N2‐4	1.3	0.7–2.2	0.44	1.1	0.6–1.9	0.84	1.1	1.2–1.8	0.63

Active CD had no significant change in the number of γδ IELs or median ratio of γδ/CD3, compared to inactive CD. In contrast, active UC had a significant loss of γδ IELs and reduction in the ratio of γδ/CD3 IELs compared to inactive UC (both *p* < 0.001). Both CD and UC had fewer γδ IELs (*p* = 0.06, *p* = 0.002) and lower γδ/CD3 IEL ratios (*p* = 0.001, *p* < 0.001) than HC (Table 3, Figure 3A–D).

#### Active CD and UC have constant numbers of γδ T cells in subepithelium and lamina propria and a significant increase of CD3 cells

Active CD and UC had an increase in subepithelial (*p* = 0.046, *p* = 0.005) and lamina propria CD3 cells (*p* = 0.002, *p* < 0.001) compared to inactive disease and HC (all *p* < 0.001) (Table 3, Figure 2A–C,E,F). The numbers of γδ T cells beneath the epithelium and in lamina propria remained constant (Table 3, Figure 3A–C,E,F). The subepithelial and lamina propria γδ/CD3 ratios were lower in active disease due to increased numbers of CD3 cells. This difference in ratio was significant for active versus inactive subepithelium for UC (*p* = 0.001) and in lamina propria for both CD (*p* = 0.037) and UC (*p* = 0.004) (Table 3). The ratios were also significantly lower for active UC (*p* = 0.02) and nearly for CD (*p* = 0.09) compared to HC (Table 3).

### CD3 and γδ T cells in mucosal compartments in relation to clinical and histological variables

#### Proximal colon has higher numbers of mucosal γδ T and CD3 cells than distal colon

Comparing cell numbers between proximal and distal colon mucosal compartments across all groups showed more CD3 (Figure 2D–F) and γδ T cells (Figure 3D–F) proximally. The difference was significant for CD3 IELs (FC 1.4, *p* = 0.008) and subepithelially (FC 1.3, *p* = 0.019), but not in lamina propria (FC 1.2, *p* = 0.16). The difference was also significant for γδ IELs (FC 1.5, *p* = 0.03) and in lamina propria (FC 1.4, *p* = 0.007), and nearly for subepithelial γδ T cells (FC 1.5, *p* = 0.06). The γδ/CD3 ratios were similar in proximal and distal colon mucosa.

To avoid bias from changes caused by active disease, separate comparisons were made only for HC and inactive IBD. The number of samples was too low in the inactive CD and UC groups to draw conclusions. When merged into one inactive IBD group, we found significantly more γδ T cells in proximal lamina propria, but this was not the case for CD3 cells. HC clearly had more CD3 and γδ T cells within and beneath the epithelium proximally, while the numbers in lamina propria were similar (data not shown).

#### Oral corticosteroids impact the number of γδ T cells, but not CD3 cells, while age, gender, and disease duration have no impact

Oral corticosteroids had a significant impact on γδ T cell numbers, but not the CD3 cell numbers. We found fewer γδ T cells in the epithelium (*p* = 0.015), subepithelium (*p* = 0.001), and lamina propria (*p* = 0.017) in patients using this medication. Adjusted for oral corticosteroids, the significant differences already found between the groups became stronger and showed clearer evidence of more intraepithelial (FC 1.7 [1.0–2.8], *p* = 0.049) and subepithelial (FC 1.7 [1.0–2.8], *p* = 0.046) γδ T cells in inactive UC compared to HC (Table 2). Age, gender, and disease duration did not impact the number of CD3 or γδ T cells in the mucosal compartments.

#### There is no correlation between the ratio of γδ/CD3 T cells in peripheral blood and colon mucosa in HC or during inflammation in active IBD

As we found significant differences in γδ/CD3 IEL ratios between the groups, flow cytometric measurements were done for a subset of patients with active disease (UC, *n* = 19; CD, *n* = 13; HC, *n* = 15) to see if these differences correlated to the number of γδ T cells in peripheral blood. There were statistically similar median (95% CI) γδ/CD3 ratios in peripheral blood between the groups, with 2.9 (1.4–4.4) in CD, 2.8 (1.0–4.2) in UC, and 3.5 (1.5–6.6) in HC. Spearman correlation analyses between the γδ/CD3 ratios in blood and in the mucosal compartments showed no significant correlations, with a moderate rho of 0.41 (−0.14 to 0.77) for the subepithelial compartment in HC as the strongest.

## Discussion

This study used AI‐based image analysis on WSIs [31] to describe the number and distribution of CD3 and γδ T cells in colon mucosa and reveals significant differences between CD and UC. At baseline, we show less γδ IELs and more CD3 cells in all mucosal compartments in inactive CD compared to inactive UC and HC. In active disease, there was no increase of CD3 IELs in CD or UC, despite a substantial increase in the subepithelium and in lamina propria. The number of γδ T cells also remained constant in the subepithelium and lamina propria. Our most important findings were consistently low numbers of γδ IELs in CD contrasting a significant loss of γδ IELs in active UC.

The literature reports conflicting results on the number of γδ T cells in colon mucosa in IBD [22, 36, 37, 38, 39, 40]. It is difficult to compare studies when histological descriptions are lacking, methods and biopsy location vary, and mucosal compartments are not always distinguished. Fukushima *et al* [25] described greater reduction of γδ IELs in inflamed UC than CD, like us. A flow cytometry study by Jaeger *et al* [40] showed a reduction of γδ IELs in both inactive and active CD, but they looked at terminal ileum. We have not found studies that report consistently low numbers of γδ IELs in colon mucosa of inactive and active CD, like we do here.

We show an association between oral corticosteroids and significantly reduced numbers of γδ T cells in all compartments, while age, gender, and disease duration had no impact on the γδ T cells numbers. When adjusting for oral corticosteroids in the statistical analysis, the differences in γδ IELs between CD and UC became even more pronounced.

The massive loss of γδ IELs in active UC made us question their fate. Our study is merely descriptive, but we tried to answer some of the questions based on what we could observe. We asked whether γδ IELs in active UC were expelled with ulcer exudates but found a significant reduction of these cells also in active inflammation without ulcerations. They might have escaped the epithelium, but we found no increase of γδ T cells beneath the epithelium or in lamina propria. They could be involved in the shedding of epithelial cells, but lymphocytes are hardly never seen above the epithelial surface. Interestingly though, Hu *et al* recently describe that γδ IELs facilitate shedding of epithelial cells [20]. We also asked if γδ IELs in active UC might exit the mucosa and enter the blood stream and made correlations between γδ/CD3 ratios in mucosal compartments and blood. No association was found, suggesting there is no significant exchange of γδ T cells between peripheral blood and tissue [21, 22].

Another interesting finding in our study is the higher numbers of CD3 cells in all compartments in inactive CD compared to inactive UC and HC. We have not found other studies that investigate the mucosal numbers of CD3 cells in inactive IBD, measure the magnitude of increase in active disease, or explore the IELs on WSIs from IBD patients. The subepithelial area was delineated by us and is not a morphologically defined zone but were included as a separate analysis because we observed noticeable variations in this zone. We believe the subepithelial lymphocytes may form an unrecognised part of the mucosal barrier together with epithelial cells, IELs, and other immune cells in the upper part of lamina propria. We did not expect to find other studies that specifically describe subepithelial T cells. However, an *in situ* hybridisation study by Kappeler *et al* [41] showed a higher increase of cytotoxic T cells in lamina propria in CD than in UC, with cytotoxic T cells located close to the epithelium. It is well known that there are more IELs above lymphoid aggregates and follicles, but we did not compare the number of organised lymphoid structures in CD, UC, and HC. We observed no obvious differences between the groups, but this clearly needs to be analysed more objectively.

We found no increase of CD3 IELs in active CD and UC, despite a significant increase of subepithelial and lamina propria CD3 cells. It is not clear if subepithelial CD3 cells are stationary or move between the compartments, or if IELs self‐renew *in situ* or are replaced by new T cells from the circulation. An argument for the latter is that thin and elongated lymphocytes crossing the basement membrane are easily seen histologically [42, 43]. Knowledge about IELs is largely derived from studies on mouse models [9], and species differences may be important. Ahn *et al* [44] reported an increase in colonic IELs in inflamed UC, but most human studies describe no change in the number of IELs in inflamed colon mucosa of IBD [24, 25, 45]. Fiehn *et al* [46] used a commercial digital image analysis tool to quantify CD3 IELs in microscopic colitis, and they and others describe IEL numbers [13, 24, 25] and γδ/CD3 IEL ratios [13, 25, 39] comparable to us.

An important question arising from our study is why colon mucosa in inactive CD has more CD3 cells and less γδ IELs than HC and inactive UC. As alterations in the number of γδ IELs may influence mucosal immunity, our findings should be further investigated in studies that also integrate disease outcomes. One may assume that a reduction of these cells could lead to increased susceptibility to infections and relapses and alter other immune cells in the epithelial area. The increased numbers of subepithelial CD3 cells in our CD patients could fit with the latter. Additionally, we found more CD3 and γδ T cells in proximal colon mucosa, which also has a higher bacterial density [47]. Since proximal colon is a predilection site for CD, it seems reasonable that fewer γδ IELs combined with increased bacterial challenge could be a contributive factor to CD pathogenesis. Our findings may support the hypothesis that CD represents a deficiency state of innate immunity [48, 49, 50]. It has been shown that γδ IELs release cytokines, chemokines, and survival signals that attract and stimulate neutrophils [51]. We hypothesise that fewer γδ IELs negatively influence the recruitment and function of neutrophils and place a higher strain on other immune cells causing deeper inflammation in the gut wall [48].

There are some limitations to our study. The number of patients could have been higher and more equal between UC and CD, and it would be better to have segmental biopsies instead of the more limited cases from our in‐house biobank with healthy and inflamed mucosa only. The IHC staining was done manually on subsequent sections which led to more variations between the images. Some mis‐quantifications may have occurred due to weak CD3 staining combined with background staining in the epithelium in some sections. Thus, we examined all WSIs in two extra rounds and agreed upon which to include based on IHC quality. Although we did not find systematic differences in the orientation of biopsies, it is possible that variations in cut surfaces may have influenced the results. Some sections have less surface epithelium, or lack it altogether, and in these cases the intraepithelial counts reflect mainly crypt epithelial lymphocytes. This may represent a confounder as it is currently unknown whether there is a difference in the number of IELs between surface and crypt epithelium.

Research on mucosal inflammation in IBD often centres on isolated molecules or cells, detached from their spatial relationships. Computational pathology provides quantitative data while preserving the histopathological contexts of cells and tissues. The key strengths of this study lie in the automated segmentation and immune cell quantifications on WSIs from a cohort of well‐characterised IBD patients, using proximal and distal colon biopsies and a validated histological index. The study reveals significant differences in CD3 and γδ T cells in the epithelial area between CD, UC, and HC. We believe that the combination of quantitative data with precise histological descriptions can elucidate some of the dynamics of these important cells in the mucosal immune responses.

## Author contributions statement

ESR, HPSP, AKS, IC‐S and IB contributed to the conception and design of different parts of the study. ESR and HPSP performed the histopathological experiments and training of the AI‐based algorithms and WX, AL and IC‐S performed the PBMC analysis. ESR, HPSP, WX, AL, AKS, SES, IC‐S and IB contributed to the assembly, analysis, and interpretation of data. ERS and IB wrote the manuscript, and all authors critically revised and approved the final version.

## Data Availability

The dataset of 251 (HE and CD3‐stained) colon biopsy WSIs used for developing the deep learning‐based model for epithelial segmentation is made openly available at DataverseNO (https://doi.org/10.18710/TLA01U). All source code and a tutorial video can be found in the GitHub repository (https://github.com/andreped/NoCodeSeg). The other datasets used and analysed in the current study are available from the corresponding author on reasonable request.

## References

[1] Burisch J , Jess T , Martinato M , *et al*. The burden of inflammatory bowel disease in Europe. J Crohns Colitis 2013; 7: 322–337. 23395397 10.1016/j.crohns.2013.01.010

[2] Catalan‐Serra I , Brenna O . Immunotherapy in inflammatory bowel disease: novel and emerging treatments. Hum Vaccin Immunother 2018; 14: 2597–2611. 29624476 10.1080/21645515.2018.1461297 PMC6314405

[3] Mosli MH , Parker CE , Nelson SA , *et al*. Histologic scoring indices for evaluation of disease activity in ulcerative colitis. Cochrane Database Syst Rev 2017; 5: CD011256. 28542712 10.1002/14651858.CD011256.pub2 PMC6481362

[4] Novak G , Parker CE , Pai RK , *et al*. Histologic scoring indices for evaluation of disease activity in Crohn's disease. Cochrane Database Syst Rev 2017; 7: CD012351. 28731502 10.1002/14651858.CD012351.pub2 PMC6483549

[5] Lang‐Schwarz C , Agaimy A , Atreya R , *et al*. Maximizing the diagnostic information from biopsies in chronic inflammatory bowel diseases: recommendations from the Erlangen International Consensus Conference on Inflammatory Bowel Diseases and presentation of the IBD‐DCA score as a proposal for a new index for histologic activity assessment in ulcerative colitis and Crohn's disease. Virchows Arch 2021; 478: 581–594. 33373023 10.1007/s00428-020-02982-7 PMC7973393

[6] Lang‐Schwarz C , Angeloni M , Agaimy A , *et al*. Validation of the “inflammatory bowel disease – distribution, chronicity, activity (IBD‐DCA) score” for ulcerative colitis and Crohn's disease. J Crohns Colitis 2021; 15: 1621–1630. 33773497 10.1093/ecco-jcc/jjab055 PMC8495487

[7] Jouret‐Mourin A , Faa G , Geboes K . Colitis (2nd edn). Cham: Springer Nature, 2018.

[8] de Souza HS , Fiocchi C . Immunopathogenesis of IBD: current state of the art. Nat Rev Gastroenterol Hepatol 2016; 13: 13–27. 26627550 10.1038/nrgastro.2015.186

[9] Hu MD , Edelblum KL . Sentinels at the frontline: the role of intraepithelial lymphocytes in inflammatory bowel disease. Curr Pharmacol Rep 2017; 3: 321–334. 29242771 10.1007/s40495-017-0105-2 PMC5724577

[10] Larmonier CB , Shehab KW , Ghishan FK , *et al*. T lymphocyte dynamics in inflammatory bowel diseases: role of the microbiome. Biomed Res Int 2015; 2015: 504638. 26583115 10.1155/2015/504638 PMC4637034

[11] Ma H , Tao W , Zhu S . T lymphocytes in the intestinal mucosa: defense and tolerance. Cell Mol Immunol 2019; 16: 216–224. 30787416 10.1038/s41423-019-0208-2 PMC6460495

[12] Kaser A , Zeissig S , Blumberg RS . Inflammatory bowel disease. Annu Rev Immunol 2010; 28: 573–621. 20192811 10.1146/annurev-immunol-030409-101225 PMC4620040

[13] Lutter L , Hoytema van Konijnenburg DP , Brand EC , *et al*. The elusive case of human intraepithelial T cells in gut homeostasis and inflammation. Nat Rev Gastroenterol Hepatol 2018; 15: 637–649. 29973676 10.1038/s41575-018-0039-0

[14] Van Kaer L , Olivares‐Villagómez D . Development, homeostasis, and functions of intestinal intraepithelial lymphocytes. J Immunol 2018; 200: 2235–2244. 29555677 10.4049/jimmunol.1701704 PMC5863587

[15] Edelblum KL , Shen L , Weber CR , *et al*. Dynamic migration of γδ intraepithelial lymphocytes requires occludin. Proc Natl Acad Sci U S A 2012; 109: 7097–7102. 22511722 10.1073/pnas.1112519109 PMC3345021

[16] Kalyan S , Kabelitz D . Defining the nature of human γδ T cells: a biographical sketch of the highly empathetic. Cell Mol Immunol 2013; 10: 21–29. 23085947 10.1038/cmi.2012.44 PMC4003173

[17] Papadopoulou M , Sanchez Sanchez G , Vermijlen D . Innate and adaptive γδ T cells: how, when, and why. Immunol Rev 2020; 298: 99–116. 33146423 10.1111/imr.12926

[18] Vermijlen D , Gatti D , Kouzeli A , *et al*. γδ T cell responses: how many ligands will it take till we know? Semin Cell Dev Biol 2018; 84: 75–86. 29402644 10.1016/j.semcdb.2017.10.009

[19] McCallion O , Hester J , Issa F . Deciphering the contribution of γδ T cells to outcomes in transplantation. Transplantation 2018; 102: 1983–1993. 29994977 10.1097/TP.0000000000002335 PMC6215479

[20] Hu MD , Golovchenko NB , Burns GL , *et al*. γδ intraepithelial lymphocytes facilitate pathological epithelial cell shedding via CD103‐mediated granzyme release. Gastroenterology 2021; 162: 877–889. 34861219 10.1053/j.gastro.2021.11.028 PMC8881348

[21] Fischer MA , Golovchenko NB , Edelblum KL . γδ T cell migration: separating trafficking from surveillance behaviors at barrier surfaces. Immunol Rev 2020; 298: 165–180. 32845516 10.1111/imr.12915 PMC7968450

[22] Catalan‐Serra I , Sandvik AK , Bruland T , *et al*. Gammadelta T cells in Crohn's disease: a new player in the disease pathogenesis? J Crohns Colitis 2017; 11: 1135–1145. 28333360 10.1093/ecco-jcc/jjx039

[23] Catalan‐Serra I , Andreu‐Ballester JC , Bruland T , *et al*. Gammadelta T cells: unconventional T cells involved in IBD pathogenesis. Dig Dis Sci 2018; 63: 1977–1979. 29752622 10.1007/s10620-018-5059-7

[24] Hirata I , Berrebi G , Austin LL , *et al*. Immunohistological characterization of intraepithelial and lamina propria lymphocytes in control ileum and colon and in inflammatory bowel disease. Dig Dis Sci 1986; 31: 593–603. 2423309 10.1007/BF01318690

[25] Fukushima K , Masuda T , Ohtani H , *et al*. Immunohistochemical characterization, distribution and ultrastructure of lymphocytes bearing the gamma/delta T‐cell receptor in the human gut. Virchows Arch B Cell Pathol Incl Mol Pathol 1991; 60: 7–13. 1673280 10.1007/BF02899521

[26] van der Laak J , Litjens G , Ciompi F . Deep learning in histopathology: the path to the clinic. Nat Med 2021; 27: 775–784. 33990804 10.1038/s41591-021-01343-4

[27] Srinidhi CL , Ciga O , Martel AL . Deep neural network models for computational histopathology: a survey. Med Image Anal 2021; 67: 101813. 33049577 10.1016/j.media.2020.101813 PMC7725956

[28] Gubatan J , Levitte S , Patel A , *et al*. Artificial intelligence applications in inflammatory bowel disease: emerging technologies and future directions. World J Gastroenterol 2021; 27: 1920–1935. 34007130 10.3748/wjg.v27.i17.1920 PMC8108036

[29] Marchal‐Bressenot A , Salleron J , Boulagnon‐Rombi C , *et al*. Development and validation of the Nancy histological index for UC. Gut 2017; 66: 43–49. 26464414 10.1136/gutjnl-2015-310187

[30] Marchal‐Bressenot A , Scherl A , Salleron J , *et al*. A practical guide to assess the Nancy histological index for UC. Gut 2016; 65: 1919–1920. 10.1136/gutjnl-2016-312722 PMC5099187 27566129

[31] Pettersen HS , Belevich I , Røyset ES , *et al*. Code‐free development and deployment of deep segmentation models for digital pathology. Front Med (Lausanne) 2021; 8: 816281. 35155486 10.3389/fmed.2021.816281 PMC8829033

[32] Bankhead P , Loughrey MB , Fernández JA , *et al*. QuPath: open source software for digital pathology image analysis. Sci Rep 2017; 7: 16878. 29203879 10.1038/s41598-017-17204-5 PMC5715110

[33] Belevich I , Joensuu M , Kumar D , *et al*. Microscopy image browser: a platform for segmentation and analysis of multidimensional datasets. PLoS Biol 2016; 14: e1002340. 26727152 10.1371/journal.pbio.1002340 PMC4699692

[34] Belevich I , Jokitalo E . DeepMIB: user‐friendly and open‐source software for training of deep learning network for biological image segmentation. PLoS Comput Biol 2021; 17: e1008374. 33651804 10.1371/journal.pcbi.1008374 PMC7954287

[35] Schmidt U , Weigert M , Broaddus C , *et al*. Cell detection with star‐convex polygons. In: Medical Image Computing and Computer Assisted Intervention – MICCAI 2018. Lecture Notes in Computer Science, Volume 11071, Frangi A , Schnabel J , Davatzikos C , *et al*. (Eds). Cham: Springer, 2018; 265–273.

[36] Fukushima K , Masuda T , Ohtani H , *et al*. Immunohistochemical characterization, distribution, and ultrastructure of lymphocytes bearing T‐cell receptor γ/δ in inflammatory bowel disease. Gastroenterology 1991; 101: 670–678. 1860632 10.1016/0016-5085(91)90524-o

[37] Kadivar M , Petersson J , Svensson L , *et al*. CD8αβ+ γδ T cells: a novel T cell subset with a potential role in inflammatory bowel disease. J Immunol 2016; 197: 4584–4592. 27849165 10.4049/jimmunol.1601146

[38] Trejdosiewicz LK , Calabrese A , Smart CJ , *et al*. Gamma delta T cell receptor‐positive cells of the human gastrointestinal mucosa: occurrence and V region gene expression in *Heliocobacter pylori*‐associated gastritis, coeliac disease and inflammatory bowel disease. Clin Exp Immunol 1991; 84: 440–444. 1828397 PMC1535443

[39] McVay LD , Li B , Biancaniello R , *et al*. Changes in human mucosal gamma delta T cell repertoire and function associated with the disease process in inflammatory bowel disease. Mol Med 1997; 3: 183–203. 9100225 PMC2230043

[40] Jaeger N , Gamini R , Cella M , *et al*. Single‐cell analyses of Crohn's disease tissues reveal intestinal intraepithelial T cells heterogeneity and altered subset distributions. Nat Commun 2021; 12: 1921. 33771991 10.1038/s41467-021-22164-6 PMC7997960

[41] Kappeler A , Mueller C . The role of activated cytotoxic T cells in inflammatory bowel disease. Histol Histopathol 2000; 15: 167–172. 10668207 10.14670/HH-15.167

[42] Toner PG , Ferguson A . Intraepithelial cells in the human intestinal mucosa. J Ultrastruct Res 1971; 34: 329–344. 4323025 10.1016/s0022-5320(71)80076-x

[43] Ferguson A . Intraepithelial lymphocytes of the small intestine. Gut 1977; 18: 921–937. 338444 10.1136/gut.18.11.921 PMC1411743

[44] Ahn JY , Lee KH , Choi CH , *et al*. Colonic mucosal immune activity in irritable bowel syndrome: comparison with healthy controls and patients with ulcerative colitis. Dig Dis Sci 2014; 59: 1001–1011. 24282051 10.1007/s10620-013-2930-4

[45] Selby WS , Janossy G , Bofill M , *et al*. Intestinal lymphocyte subpopulations in inflammatory bowel disease: an analysis by immunohistological and cell isolation techniques. Gut 1984; 25: 32–40. 6228498 10.1136/gut.25.1.32 PMC1432224

[46] Fiehn AK , Clausen LN , Engel U , *et al*. Establishment of digital cutoff values for intraepithelial lymphocytes in biopsies from colonic mucosa with lymphocytic colitis. Pathol Res Pract 2019; 215: 152580. 31522788 10.1016/j.prp.2019.152580

[47] Arnoldini M , Cremer J , Hwa T . Bacterial growth, flow, and mixing shape human gut microbiota density and composition. Gut Microbes 2018; 9: 559–566. 29533125 10.1080/19490976.2018.1448741 PMC6287699

[48] Segal AW . The role of neutrophils in the pathogenesis of Crohn's disease. Eur J Clin Invest 2018; 48: e12983. 29931668 10.1111/eci.12983

[49] Marks D , Segal A . Innate immunity in inflammatory bowel disease: a disease hypothesis. J Pathol 2008; 214: 260–266. 18161747 10.1002/path.2291 PMC2635948

[50] Vinh DC , Behr MA . Crohn's as an immune deficiency: from apparent paradox to evolving paradigm. Expert Rev Clin Immunol 2013; 9: 17–30. 23256761 10.1586/eci.12.87

[51] Sabbione F , Gabelloni ML , Ernst G , *et al*. Neutrophils suppress gammadelta T‐cell function. Eur J Immunol 2014; 44: 819–830. 24271816 10.1002/eji.201343664

